# Understanding Children’s Online Victimization through the Psychosocial Lens: The Roles of Loneliness, Online Social Currency, and Digital Citizenship

**DOI:** 10.3390/healthcare12010097

**Published:** 2023-12-31

**Authors:** Yi-Ping Hsieh, Bonni Gourneau

**Affiliations:** 1 Department of Social Work, College of Nursing and Professional Disciplines, University of North Dakota, Grand Forks, ND 58202, USA; 2 Department of Teaching, Leadership and Professional Practice, College of Education and Human Development, University of North Dakota, Grand Forks, ND 58202, USA; bonni.gourneau@und.edu

**Keywords:** online victimization, loneliness, social currency, digital citizenship, online activities, online social capital

## Abstract

This study employed a risk and resilience framework to investigate the influence of multidimensional factors, considering psychosocial and behavioral aspects, on online victimization among fifth-grade children (ages 10–11). Loneliness, online social currency disturbance, and digital citizenship were examined as predictors of online victimization. Data were collected from 196 students through a self-reported online survey conducted on electronic devices provided by the schools. The findings indicated that 78.6% of fifth-graders owned a smartphone, 70.9% had a gaming console, and the most common online activities were playing online gaming (73%), talking with friends (62.8%), and seeking entertainment (62.2%). Online victimization was prevalent, with 30.8% of children reporting they had been called bad names, 24.7% receiving rude comments, 15.9% expressing feelings of worry or threat due to online harassment, and 3.1% experiencing cyberbullying lasting for days. Furthermore, the results revealed a negative association between digital citizenship and online victimization, while loneliness and online social currency disturbance were positively associated with online victimization after accounting for children’s gender and time spent online. In conclusion, this study suggests that efforts to prevent and address online victimization should prioritize promoting digital citizenship and increasing awareness of the roles of loneliness and social currency disturbances in online social dynamics.

## 1. Introduction

### 1.1. Online Victimization

As society becomes increasingly dependent on modern technology, children gain greater access to and exposure to the Internet, smartphones, tablets, electronic gaming consoles, and various digital devices at younger ages. While smartphones and tablets are often used as soothing tools to distract children from distress [1], they also render them more vulnerable and susceptible to internet addiction [2] and online victimization, such as cyberbullying [3], online harassment, and unwanted sexual solicitation [4], as they spend more time on and access more platforms in the cyber world. In this digital age, concerns about online victimization and the digital well-being of children have become paramount for parents. Most research concerning online victimization has been conducted with samples of adolescents, college students, and adults [5,6,7]. Less is known about the prevalence and nature of online victimization among elementary school children and the examination of its risk factors from psychosocial and behavioral perspectives. The current study aims to examine the roles of loneliness, online social currency, and digital citizenship relating to children’s experiences of online victimization.

Online harassment, cyberbullying, and exposure to unwanted sexual materials and solicitation have been reported by children as major types of online victimization [8]. Online harassment refers to an intentional and overt act of aggression toward another person online, such as making rude or nasty comments or intentionally embarrassing someone in retaliation. The prevalence of online harassment was 11% among youth and was more likely to come from a school friend and occur on a social networking site [9]. Cyberbullying refers to “an aggressive act or behavior that is carried out using electronic means by a group or an individual repeatedly and over time against a victim who cannot easily defend himself or herself” [10]. One study found that 10% of 11-year-old children from 42 countries have experienced cyberbullying via messages at least once in the past year [11]. Other studies in the United Kingdom revealed that 23% to 51% of children (ages 7 to 11) reported being victims of cyberbullying [12,13]. Moreover, unwanted sexual solicitation refers to the act of encouraging someone to talk about sex, to do something sexual, or to share personal sexual information even when that person does not want to [14]. The prevalence of being a target of unwanted online sexual solicitation among children and adolescents (aged 10 to 17) in the United States was around 19% in 2000 and 9% in 2010 [15], and was 15.4% in Taiwan in 2020 [4].

### 1.2. Multidimensional Factors in Online Victimization

According to Routine Activity Theory [16], victimization typically requires three key components: the presence of suitable targets, the absence of capable guardians, and the presence of motivated offenders. Factors such as being children, loneliness, excessive online activities, and a strong desire for social media validation (e.g., having many “likes”) can make them vulnerable targets for online harassment and cyberbullying. Furthermore, the characteristics of the internet, including anonymity and the ease of republishing and distributing content, make it challenging for parents, teachers, and other guardians to supervise or control online interactions and messages effectively. While guardians may not always be physically present, there is an opportunity to instill responsible digital behavior, often called “digital citizenship”, which can guide children in navigating the online world safely and responsibly. It is possible that children often place trust in what they encounter online and make themselves easily accessible to others, and many commonly seek new online friendships without much hesitation or precaution, especially when experiencing loneliness. This trust can increase their vulnerability to motivated online offenders or perpetrators. Based on these components of victimization occurrences, we included loneliness, online social currency disturbance, digital citizenship, and time spent online as predictors of online victimization as a lens to understand risk factors from psychosocial and behavioral perspectives.

Loneliness refers to unpleasant and distressing feelings when individuals perceive deficiencies in their social and interpersonal relationships [17]. Loneliness stems from subjective assessments of social acceptance and satisfaction with social relationships rather than the number of friends or frequency of contacts [18,19]. Previous research has established a link between loneliness and various mental health issues, including perceived stress, anxiety, anger, and reduced self-esteem [20,21], as well as externalizing problems such as delinquency and school dropout [18,22]. Furthermore, in the digital age, loneliness has become intertwined with problematic social media usage [23]. Research found that higher levels of social connectedness are associated with lower levels of loneliness [24]. As individuals strive to alleviate their loneliness, they often turn to the cyberworld to increase their social connections, finding it less daunting and more convenient to establish connections behind screens. When experiencing loneliness, individuals tend to seek comfort, emotional connection, and support in the virtual realm, leading to heightened engagement in social media and online activities. However, the heightened online presence also elevates the risk of online victimization. Research has revealed a strong correlation between elevated levels of loneliness and cyber-victimization among adolescents and young adults [25,26].

Social currency, like traditional money, serves as a means to assign value to a product or service. Within the realm of social media, these “likes”, comments, and shares have evolved into a form of online social currency through which we evaluate the worth of something [27], or, in this case, the worth of someone. This phenomenon is often referred to as the “economy of attention”. In the marketing world, everything vies for attention. When a person assigns a “like” or a portion of their limited attention to something, it essentially constitutes a recorded transition, attributing value. This is advantageous when you are marketing products such as clothing or cars. However, the issue arises when it comes to social media platforms, where we are the “product” [27]. We permit others to determine our value. We may take our product (e.g., photos) off the digital shelf because it did not sell quickly enough (i.e., it did not garner as many likes as anticipated). This behavior is reshaping our sense of identity [27]. We are attaching self-worth to external judgments online, quantifying it for public consumption, and becoming fixated on the process. People strive to look good, smart, or knowledgeable in front of others, both online and offline. Online social currency occurs when individuals compete for others’ attention and likes. Berger [28] revealed that content containing information that is unusual, extraordinary, or challenges our expectations tends to be shared and discussed more frequently. Similarly, Berger and Iyengar [29] found that the more interesting and exciting the content, the more likely it is to be shared. Such content stands out when it is unexpected, mysterious, controversial, or exciting. Consequently, in pursuit of more online social currency, it is possible that young individuals may share extraordinary personal information or images online, which can inadvertently compromise their online privacy and make them vulnerable to online victimization. When children care so much about how people think about them online, they are more susceptible to manipulation and online victimization. While the social currency has been widely employed in marketing, its application to comprehending online victimization is relatively novel. Similar to marketing products, individuals compete for attention and likes on social media platforms. Consequently, the concept of social currency can provide insights into the dynamics of online interactions and their impact on mental health and online behaviors.

Beyond social and psychological factors (i.e., loneliness and online social currency disturbance), our exploration extends to protective factors against online victimization from a behavioral perspective, specifically focusing on digital citizenship. A conceptual debate surrounding digital citizenship has unfolded, encompassing diverse definitions, theoretical frameworks, and assessment methodologies [30]. Distinguishing from digital literacy (i.e., computer and internet-based skills), digital citizenship refers to appropriate, safe, and responsible behavior norms, including critical thinking and ethical decision-making in the context of technology use [31,32]. Ribble and Miller [33] proposed a framework that categorizes nine elements of digital citizenship into three dimensions: respect yourself/respect others, educate yourself/connect with others, and protect yourself/protect others. Conversely, Jones and Mitchell [31] proposed a two-dimensional model of digital citizenship, encompassing online respect and online civic engagement, while Choi et al. [34] introduced a five-dimensional perspective. Digital citizenship emphasizes practicing positive social skills online and engaging with their online communities. Unlike fear-based strategies that focus solely on the harms of cyberbullying, digital citizenship emphasizes the importance of respectful behavior, perspective-taking, conflict resolution strategies, and tolerance for diverse viewpoints when navigating disagreements online [31]. The digital citizenship curriculum offered by Common Sense Media is the most widely adopted program in 76% of public schools across the United States (www.comomsense.org, accessed on 25 October 2023). This curriculum covers essential topics such as internet safety, privacy and security, information literacy, cyberbullying, and digital drama. While prior research has primarily focused on defining the concepts and components and measuring digital citizenship [30,35], as well as exploring digital citizenship education [36], our understanding of the linkages between digital citizenship and online victimization, particularly among children and adolescents, remains limited. Only a handful of studies have delved into the relationship between digital citizenship and cyberbullying perpetration behaviors in college students [37]. Establishing evidence-based connections between digital citizenship and online victimization is essential, as is examining whether digital citizenship can effectively reduce the risk of online victimization.

### 1.3. The Current Study

This current study utilizes a risk and resilience framework [38] to examine the potential risk factors (loneliness and online social currency disturbance) and protective factors (digital citizenship) as related to online victimization among children. Moreover, we explored these factors based on the key components of Routine Activity Theory (e.g., the presence of suitable targets and the presence of motivated offenders) in studying victimization. We hypothesized that loneliness and strong desire and disturbance for social currency increased the risks of being vulnerable and suitable targets, while appropriate, safe, and responsible online behaviors (digital citizenship) reduced the risks of motivating online perpetrators. Consequently, this proactive approach assists in reducing the overall risks of online victimization. Overall, the ultimate goal is to enable children to benefit from cyber systems while reducing their risks of cyber victimization by raising awareness, incentivizing behavioral changes, and helping with decision-making processes regarding online behaviors. The first effort of this study investigated popular social media applications among children, online activities they primarily engaged in, online victimization they experienced, and online behaviors children may engage in that put their online safety at risk. The second goal is to identify the multidimensional risk factors for predicting online victimization from psychosocial and behavioral perspectives. This project enhances our knowledge about how multidimensional factors affect the risks of online victimization among children.

## 2. Materials and Methods

### 2.1. Participants

A total of 196 fifth graders (79% girls) from 15 classes in five public elementary schools in North Dakota participated in this study. Parents provided consent, and children provided assent. Children were 10.83 years old (SD = 0.43) on average. Most children resided in two-parent homes (81%). Most children have either one (43%) or two (28%) siblings living with them; some do not have any siblings (13%); and some have three to six siblings (14%) residing within their homes. Most of them lived in either a single house or townhouse (80%), some in apartments (16%), and only a few in other housing, such as a trailer (3%).

### 2.2. Procedure

Upon obtaining approval from the superintendent of schools, we sent an introductory letter describing this study to seven public elementary school principals within the district. Of these, five schools agreed to participate. Subsequently, the school principals provided the names and contact information of 15 fifth-grade teachers, who all showed keen interest in this study and offered assistance in distributing and collecting parental consent and scheduling class time for the online survey. Quantitative data were collected through an online survey conducted in each of the participating classes. The researchers visited the classrooms during scheduled class time and, with the assistance of the teachers, facilitated the online survey using electronic devices provided by the schools. Students took 15–20 min to complete the online survey.

### 2.3. Measures

**Demographic information.** In addition to age and gender, children reported numbers of siblings, family members who resided together, and housing. Since the student populations are not very diverse in this town, with the majority being white, the researchers did not ask students’ ethnicity to protect their confidentiality.

**Accessibility to electronic devices.** Students were asked about the device(s) they usually use to get online, the ownership of the device(s), social media and social networking application(s) they usually use, the time they spend online, and the activities they often do online in multiple-choice and open-ended survey formats.

**Online victimization.** Online victimization was measured by eight questions adapted from previous studies [9,39]. These questions included the following: being called rude/bad names or comments online; being bullied over a few days or weeks; feeling worried or threatened because someone was bothering you online; and sending you pictures that made you feel uncomfortable. Students responded to the questions in a Yes/No matrix (0 = no, 1 = yes). The sum of these eight items computed the score. The scale used in this study demonstrated good internal consistency, with a Cronbach’s alpha (α) coefficient of 0.75.

**Loneliness**. Loneliness was measured using five items from the UCLA Loneliness Scale-Short Form (ULS-8) [40]. Minor adjustments were made in the wording of these questions to ensure the fifth graders could understand them. These items were measured on a 4-point rating scale (1 = never feel this way to 4 = often/many times feel this way). Higher scores indicate higher levels of loneliness. The scale used in this study demonstrated good internal consistency, with a Cronbach’s alpha (α) coefficient of 0.78.

**Online social currency disturbance.** Social currency, often called the economy of attention, served to ascribe value to something (e.g., produce or service). Online social currency is anything shared online because it allows others to respond to it and determine its value (e.g., through likes, comments, and shares). Based on the definition, online social currency disturbance was measured by three items. Students were asked how much they agreed on the following feelings they had: I care very much about how people like my posts or how many comments I get on my posts; I feel upset when my posts online do not get much attention; and I feel bad about myself when I see other people’s pictures and posts. The responses for all items were rated on a 5-point scale anchored by 1 (very much disagree) to 5 (very much agree). Higher scores indicate higher levels of online social currency disturbance. The scale used in this study demonstrated acceptable internal consistency, with a Cronbach’s alpha (α) coefficient of 0.70. Exploratory factor analysis was conducted to assess construct validity [41], and the results indicated strong evidence [42], with a single factor accounting for 64% of the variance and factor loadings ranging from 0.65 to 0.87.

**Digital citizenship.** Digital citizenship is defined as the norms of appropriate and responsible behavior concerning technology use. This variable was measured by eight items from the Digital Citizenship Scale [31]. Sample questions are: I watch my language online so it does not come across as mean to anyone; I do not post pictures of anyone if I think it will embarrass them or get them in trouble; I make sure I do not post anything online that I will regret later; I am confident in using the Internet to find information I need; and I am fully aware that downloading illegal music/movies is a crime and not the right thing to do. These items were measured on a 5-point rating scale (1 = very much disagree to 5 = very much agree). Higher scores indicated higher levels of digital citizenship. The scale used in this study demonstrated strong internal consistency, with a Cronbach’s alpha (α) coefficient of 0.85.

**Time spent online.** Children were asked to report how much time they spent online on a typical school day. Responses were provided on a 4-point scale, anchored by 1 (less than one hour), 2 (1 to 2 h), 3 (3 to 4 h), and 4 (more than 4 h).

## 3. Results

We employed SPSS version 28 for the statistical analyses. To gain insight into the distribution of study variables and explore their interrelationships, we initially conducted descriptive statistics. Pearson’s coefficient was utilized for correlational analysis to illustrate connections between these variables. Subsequently, a multiple linear regression analysis was carried out to assess the contribution of loneliness, online social currency disturbance, and digital citizenship to online victimization, after considering child gender and time spent online as covariates. This approach is a widely accepted method for evaluating the influence of predictor variables while accounting for the impact of other variables within the model.

Descriptive results are illustrated in Table 1. In terms of ownership of electronic devices, most 5th-grade children possess smartphones (78.6%), followed by gaming consoles (70.9%) and computers/laptops (59.2%). Regarding the accessibility and options of electronic devices, most 5th graders (73%) often use smartphones to access the internet, followed by game systems (49%) and computers/laptops (35%). The primary social media applications utilized by 5th graders were Snapchat (57.1%), followed by Instagram (40.8%), Google+ (36.2%), others such as YouTube and TikTok (26.5%), and Pinterest (20.4%). Approximately 16.8% of 5th graders do not use any social media applications. During a typical weekday, 5th graders typically spend one to two hours online (46.9%), while some spend more than four hours online (20.6%), three to four hours (17.5%), or less than one hour (14.9%). On weekends, more than half of the 5th graders (57.5%) spend more than three hours daily. In terms of online activities, most 5th graders often participated in online gaming (73%), followed by talking with friends (62.8%), seeking entertainment such as movies and music (62.2%), texting (53.6%), engaging in social media (47.4%), and browsing for educational or learning purposes (18.4%).

In terms of online victimization experiences, 30.8% of 5th graders reported being called rude or bad names online, while 24.7% indicated they have encountered rude or nasty comments written about them on the internet. Moreover, 17.9% reported being embarrassed by someone online, 16% shared that their photos were posted online by someone without permission, 15.9% felt worried or threatened due to online harassment, and 10.8% received unsettling pictures that made them feel uncomfortable. Additionally, 3.1% of children disclosed that they had experienced cyberbullying lasting for a few days, and 1.5% experienced cyberbullying for several weeks. Regarding risky online behaviors, 11.3% of 5th graders admitted to posting their personal information online, and 3.6% disclosed sending pictures of themselves to someone they met online but did not know in person. Finally, 12.3% of children admitted to making rude comments toward others online, while 9.3% admitted to playing mean jokes on someone online.

Table 2 presents the correlation coefficients for all this study variables and the variables’ means, standard deviations, and score ranges. Online victimization was positively correlated to loneliness (r = 0.28), online social currency disturbance (r = 0.27), and time spent online (r = 0.24), while it was negatively correlated to digital citizenship (r = −0.34). Table 3 summarizes the results of the multiple regression analysis of online victimization predictors. These variables significantly explained 23.6% of the variance in online victimization. The results indicated that digital citizenship was negatively associated with online victimization (β = −0.31, *p* < 0.001), while loneliness (β = 0.19, *p* = 0.004) and online social currency disturbance (β = 0.20, *p* = 0.003) were positively associated with online victimization, after accounting for children’s gender and time spent online. Children who experienced higher levels of loneliness and social currency disturbance and spent much time online were at greater risk of online victimization. In contrast, children who had higher levels of digital citizenship were at lower risk of online victimization.

## 4. Discussion

The age at which people begin using social media applications and engage in online activities has decreased. For this reason, it is important to understand children’s online experiences and examine the factors that increase their risk of being victimized online. This study confirmed the hypothesized risk and protective factors of online victimization among children from psychosocial and behavioral perspectives. The findings revealed that loneliness and online social currency disturbance increased the risk of online victimization, while digital citizenship decreased the risk.

### 4.1. Loneliness and Online Victimization

While previous studies found strong connections between loneliness and cyber-victimization among adolescents and adults [25,26], this current study revealed such a linkage among children. Loneliness often stems from an unfulfilled need for peer acceptance [43]. Loneliness typically originates from a disconnect between an individual’s desired and actual social relationships [44]. When children experience loneliness and seek peer acceptance, they often find it easier to engage in social interactions or connections in the cyber world behind screens as opposed to in the physical world. However, when children are eager to establish relationships online yet lack confidence and social skills, it can possibly heighten their vulnerability to online victimization. The results confirmed the connection between loneliness and online victimization. Educators, parents, and health professionals can foster positive social environments in classrooms, homes, and communities, providing the necessary support to combat child loneliness. This, in turn, helps reduce the risk of online victimization.

### 4.2. Online Social Currency Disturbance and Online Victimization

Furthermore, this study is pioneering in its application of the social currency concept, alongside other psychosocial factors, in understanding the dynamic of online victimization among children. Online social currency strongly emphasizes fostering relationships, including social interactions and connections with others in the digital realm. It fosters a sense of community, shapes one’s identity, facilitates access to information, and confers status and recognition [45]. However, when children use these online social currencies in the form of “likes” and comments to determine their value and self-worth, it could harm children’s self-esteem and well-being and increase their vulnerability and risk of online victimization. People typically compare themselves to individuals who possess some form of superiority (e.g., more “likes”), even in the face of potential threats to their self-esteem. These upward social comparisons often lead to a deterioration in mood and a diminished perception of one’s abilities [46]. In line with the Routine Activity Theory [16], this strong desire for social media validation and online social currency disturbance can make children vulnerable targets for online harassment and cyberbullying. While developing media education in schools, it is important not only to focus on behavior itself but also to understand the psychosocial underlying mechanisms of online behaviors. This involves being aware of how the strong desire for social currency could influence our online behaviors on social media.

### 4.3. Digital Citizenship and Online Victimization

Moreover, this study highlighted the importance of developing children’s digital citizenship competencies to prevent online victimization and equip them with tools when interacting with others online. Digital citizenship includes learning critical skills, values, and behaviors to interact with people and information online appropriately. Digital citizenship curricula were typically created in response to contemporary issues of online victimization, including cyberbullying, online safety, privacy concerns, hate speech, misinformation, and digital distraction. Instead of relying on fear-based tactics that emphasize the harm of cyberbullying, the digital citizenship curriculum prioritizes respectful conduct, perspective-taking, conflict resolution skills, and promoting tolerance for diverse viewpoints when engaging in online discussions and communications. Students are taught to make sound choices amidst the multitude of digital communication options, to independently process, analyze, and decide upon media and information, and to employ technology responsibly, becoming accountable digital citizens. Our online choices, the content we engage with, and our interactions with others can positively or negatively impact our digital well-being. While technology and the internet are meant to enrich our lives, not all online experiences benefit children and may influence their physical and mental health and social-emotional development. Through positive interaction and engagement, digital citizenship can be cultivated and instilled to mitigate children’s risky online behaviors. The results of this study confirmed the role of digital citizenship in reducing the risk of online victimization among children.

### 4.4. Limitations

While the results of the current study are promising, some limitations should be noted when interpreting the results. First, the sample of children was relatively small, limiting the ability to generalize the findings broadly. The findings can only generalize to small-sized urban districts in the Midwest. Secondly, only self-reported student data were collected. Future research should develop efficient and cost-effective methods of collecting data beyond self-report. Thirdly, the cross-sectional nature of the current study limits the ability to discern the potential causal associations among loneliness, online social currency disturbance, digital citizenship, and online victimization. Future studies would benefit from a longitudinal design. Fourthly, while social currency disturbance is a common phenomenon in the use of digital social media, the dedicated scale for its measurement has yet to be well developed and is relatively understudied. While we have assessed the reliability and construct validity of social currency disturbance, it is advisable to conduct further validity tests in future studies.

## 5. Conclusions

In conclusion, efforts to prevent and intervene in online victimization can prioritize the promotion of digital citizenship and raise awareness of the effects of loneliness and social currency disturbances on online social dynamics.

## Figures and Tables

**Table 1:** Characteristics of the Fifth-Grade Participants.

Baseline Characteristic	Participants	
	*n*	%
Gender		
Female	95	48.5
Male	101	51.5
Live with two parents		
Yes	157	80
No	39	20
Devices ownership		
Smartphone	154	78.6
Gaming Console	139	70.9
Computer/laptop	116	59.2
Primary Devices to get online		
Smartphone	143	73
Gaming Console	96	49
Computer/laptop	69	35.2
Primary social media platforms		
Snapchat	112	57.1
Instagram	80	40.8
Google+	71	36.2
YouTube or TikTok	52	26.5
Pinterest	40	20.4
Do not use social media applications	33	16.8
Time spent online in a typical weekday		
Less than one hour	29	14.9
1–2 h	91	46.9
3–4 h	34	17.5
More than four hours	40	20.6
Primary Online activities		
Online gaming	143	73
Talking with friends	123	62.8
Seeking entertainment	122	62.2
Texting	105	53.6
Engaging on social media	93	47.4
Browsing for educational purposes	36	18.4
Online victimization experiences		
Being called a bad name	60	30.8
Encountering rude/nasty comments	48	24.7
Being embarrassed by someone online	35	17.9
Personal photos were posted online by someone without permission	31	15.9
Felt worried or threatened due to online harassment	31	15.9
Receiving unsettling and uncomfortable photos	21	10.8
Being cyberbullied lasted for a few days	6	3.1
Being cyberbullied for several weeks	3	1.5
Risky online behaviors		
Posting personal information online	22	11.3
Sending personal photos to someone I met online but did not know in person	7	3.6
Making rude comments toward someone online	24	12.3
Playing mean jokes about someone online	18	9.3

Note. n = 196.

**Table 2:** Bivariate Correlations, Means, and Standard Deviations for this Study Variables.

Variables	1.	2.	3.	4.	5.
1. Online Victimization	--				
2. Loneliness	0.28 **	--			
3. Social Currency Disturbance	0.27 **	0.23 **	--		
4. Digital Citizenship	−0.34 **	−0.08	−0.06	--	
5. Time Spent Online	0.24 **	0.07	0.04	−0.18 *	--
Mean	1.21	1.82	2.30	4.34	2.44
SD	1.67	0.68	1.02	0.66	0.98
Scale range	0–8	1–4	1–5	1–5	1–4

Note. ** *p* < 0.01. * *p* < 0.05. SD is the standard deviation.

**Table 3:** Summary of Multiple Regression Analysis for Variables Predicting Online Victimization.

	Online Victimization		
Predictor Variables	B	SE B	β
Child Gender	−0.20	0.22	−0.06
Time spent online	0.28	0.11	0.16 *
Loneliness	0.46	0.16	0.19 **
Social Currency Disturbance	0.33	0.11	0.20 **
Digital Citizenship	−0.79	0.17	−0.31 ***

Notes. Codes for gender are 1 = boy, 0 = girl. * *p* < 0.05; ** *p* < 0.01; *** *p* < 0.001.

## Data Availability

Data are contained within the article.
